# Coordination and adaptation of water processes in *Populus euphratica* in response to salinity

**DOI:** 10.3389/fpls.2024.1443444

**Published:** 2024-09-06

**Authors:** Duan Li, Jianhua Si, Xiaozong Ren

**Affiliations:** ^1^ School of Geographic Sciences, Taiyuan Normal University, Jinzhong, China; ^2^ Shanxi Key Laboratory of Earth Surface Processes and Resource Ecology Security in Fenhe River Basin, Taiyuan Normal University, Jinzhong, China; ^3^ Key Laboratory of Eco-hydrology of Inland River Basin, Northwest Institute of Eco-Environment and Resources, Chinese Academy of Sciences, Lanzhou, China

**Keywords:** *Populus euphratica*, leaf-specific hydraulic conductance, water transport, water utilization, water storage, salt stress

## Abstract

Water processes secure plant survival and maintain their ecosystem function. Salinity affects water processes, but the mechanisms remain unclear and may depend on the degree of salinity stress. To improve the understanding of the cooperation of plant organs involved in water processes under salinity stress, we determined hydraulic, gas exchange, and physiological and biochemical parameters in *Populus euphratica* Oliv. under different salinity stresses. The results suggested that *P. euphratica* enhanced water transport efficiency in a salinity-stress environment, and the strengthening effect of roots in the water transfer process was greater than that of the aboveground parts. *P. euphratica* also increased water use efficiency and water transport efficiency in mild and moderate salinity stress (less than 200 mmol/L NaCl) but was adversely affected by heavy salinity stress (more than 300 mmol/L NaCl). Furthermore, *P. euphratica* increased its water storage by regulating antioxidant enzyme scavenging capacity and osmoregulation, which resulted in coordinated greater water utilization and enhanced water transport among plant organs and indicated that the adverse effects on water processes triggered by salinity stress depended on the extent of salt stress. *P. euphratica* lessened stress-induced damage and maintained plant productivity by coordination and cooperation of water processes under certain levels of salinity. Research on the coordination and cooperation involving water processes in riparian forests in saline areas provides the scientific basis for riparian plant protection and restoration.

## 1 Introduction

Salinized soils constitute 7.6% of the world’s total landmass, with significant extents of these soils in the arid northwestern regions in China. Salinity, one of the major agents of environmental disturbance, interferes with the productivity and growth of plants, especially in arid and semi-arid areas (Deinlein et al., 2014; Yang et al., 2021). Further expansion of salinized soil areas is inevitable due to continued high irrigation levels.

The arid northwestern regions of China feature downstream river basins that are well-known for expanses of riparian forests growing in oases in the desert (Chen et al., 2011; Zhang et al., 2014). *Populus euphratica*, a constructive species in oases, plays a vital role in maintaining vulnerable ecological functions in riparian areas (Chen et al., 2012). *P. euphratica* exhibits strong stress resistance and grows under adverse conditions, where it has existed for thousands of years in environments with high salinity and aridity (Si et al., 2014).

Water provision in plants relies on flow integrity through the entire water channel from roots to leaves and has significant effects on growth, function, structure, hydraulics, and ecology (Hernández et al., 2009). A decrease in water potential can be caused by both drought and salinity stress and can lead to water loss and even death of plant cells.

Water flow in plants involves the processes of water transport, utilization, and storage and affects water circulation in plants. Research on the variability in water transport efficiency provides further information on the coordination and cooperation among plant water processes and on the ecological strategy of plants to survive in detrimental environments (Pivovaroff et al., 2014). Different tissues in plants exhibit a distinct water transport ability that is dynamic and variable in the life cycle of plants (Martin et al., 2010). Past studies have shown that hydraulic conductance, which represents the ability of plants to transport water, is related to plant drought resistance (Cochard et al., 2010; Pivovaroff et al., 2016). Reducing water transport efficiency causes hydraulic limitation in woody plants. Various physiological processes, including photosynthetic production, can be constrained by hydraulic limitation caused by decreases in available water (Ambrose et al., 2010). Photosynthesis and other physiological processes may affect water use efficiency during the development of salinity stress due to a decrease in osmotic potential caused by the presence of salts in the soil solution and a reduction in water availability (Minh et al., 2016). Certain plants demonstrate improved water utilization in saline environments, whereas certain tree species display a decline in their water utilization efficiency (Li et al., 2022; Zhang et al., 2022). As a result, water utilization efficiency could be affected by water transport efficiency. Studies on the coordination between water transport efficiency and water use efficiency are necessary to understand the flexibility and adaptability of *P. euphratica* to salt-stress conditions; further, an increased understanding of the response of riparian plants to salinity gradients is crucial for ecosystem restoration.

Hydraulic limitations caused by water deficits can be compensated by utilizing water storage (Woodruff et al., 2004; Scholz et al., 2011). Enzymatic defense mechanisms, which activate antioxidant enzymes to safeguard against oxidative damage, are crucial in controlling harmful reactive oxygen species (ROS) levels, and they can affect water storage in plants (Karuppanapandian et al., 2011; AbdElgawad et al., 2016; Moukhtari et al., 2020). The formation and generation of ROS are induced in plant cells by high salinity; excess accumulation of ROS could lead to damage to membrane lipids, nucleic acids, and proteins (Gill and Tuteja, 2010; Sergio et al., 2012). Superoxide dismutase (SOD), peroxidase (POD), and catalase (CAT) are critical in enzymatic defense mechanisms and ROS elimination (Meriga, 2013; Hishida et al., 2014). Additionally, the formation of malondialdehyde (MDA) is used as an index of lipid peroxidation, the concentration of which may reflect the levels of membrane lipid peroxidation induced by polyunsaturated fatty acids under stress conditions (Zhang et al., 2014).

In addition to enzymatic defense mechanisms, osmotic adjustment, a common response to water deficits or osmotic stress, can also have a beneficial effect on water storage and compensate hydraulic limitation in plants, lowering the osmotic potential to counteract loss of turgor by means of maintaining or increasing the quantity of intra-cellular compatible solutes (Woodruff et al., 2004; Scholz et al., 2011; Moukhtari et al., 2020). Adjustment of the cell osmotic potential is required to maintain water uptake capacity, and osmoprotectants are provided by compensatory changes in compatible solutes, including amino acids, sugars, and inorganic ions (Per et al., 2017; Meena et al., 2019). Proline and soluble sugars in plants are closely connected to osmotic adjustment and stress resistance (Zelm et al., 2020). Such substances rapidly accumulate to help plants maintain certain moisture and expand pressure in cells by osmoregulation. Accumulation of inorganic ions in the cytoplasm has an essential contribution to the intra-cellular osmotic balance, which may also protect cytosolic enzymes (Bezerra-Neto et al., 2022).

Previous studies on physiological and ecological responses to drought and salinity stress indicated that *P. euphratica* has a relatively efficient drought and salinity resistance and that it formed the characteristics of adaptability to counteract the presence of salt. However, the regulation involving water processes in the salt-induced environment is still not clear, and there are few reports on the coordination among water transfer processes, water use processes, and water retention processes in *P. euphratica*. Because of its ability to survive and form forests under stressful conditions, understanding the adaptation and coordination of water transport, water utilization, and water storage in *P. euphratica* will enhance our knowledge of plant adaptation to salt-stress adaptation and our ability to restore riparian ecosystems. Here, we established a study to address: 1) How does *P. euphratica* adjust water transfer processes under different salt stress? 2) What are the changes in water transport, water utilization, and water storage processes in *P. euphratica*? 3) Are the three changes synchronized? Can they coordinate and cooperate to maintain a balance of water processes?

## 2 Materials and methods

### 2.1 Study sites

**Figure 1:**
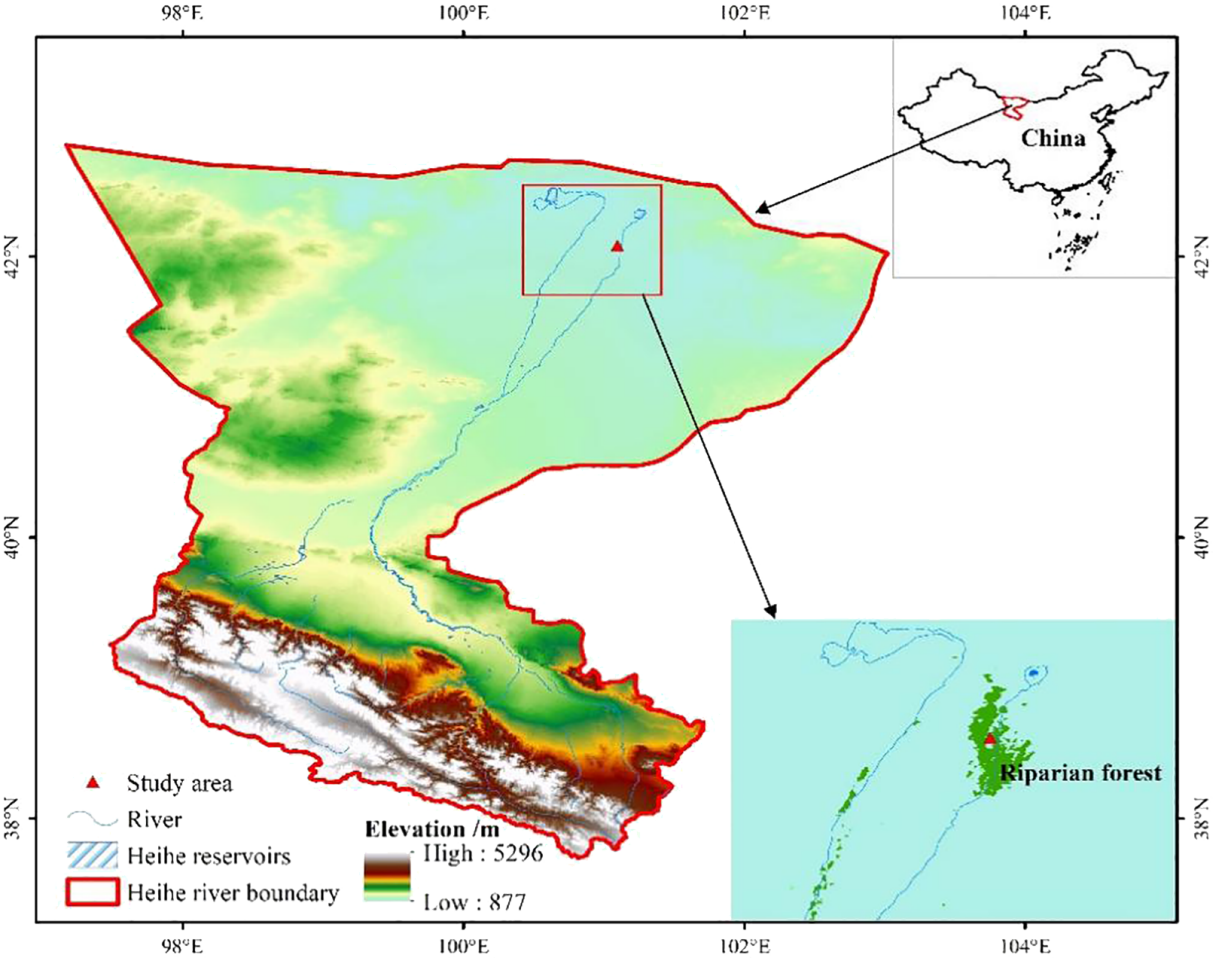
Remote sensing images of Heihe River Basin in the arid inland region of China.

The study was conducted at the Eco-Hydrology Experimental Research Station situated in the Alxa Desert, a northwestern region of China, specifically downstream of the Heihe River (42°01′N, 100°21′E) (**Figure 1**). In this region, the majority of precipitation, amounting to 75%, falls during June, July, and August, totaling an annual precipitation of 38 mm. However, evaporation is exceptionally high, reaching 3,390 mm per year, surpassing precipitation by over 90 times. Characterized by an average annual temperature of 8.2°C, this area is classified as one of the most arid regions in China, with peak temperatures typically occurring between June and August. Local groundwater serves as the primary water source for plants in this area, primarily originating from the discharge of the upper and middle reaches of the Heihe River (Zhou et al., 2013). The soil in this region originates from fluvial sediments; soil salinity can reach 33% (Si et al., 2014).

### 2.2 Plant materials

For a duration of 2 years, *P. euphratica* saplings were nurtured at the Ejina Banner’s local forest farm, before their transplantation to the designated study site. Subsequently, 100 of these saplings were transplanted into pots and placed outdoors in the natural environment during the early stages of April. Twenty well-growing, non-stressed, straight, and healthy samples (approximately 40 cm in height and 0.45 cm in diameter at breast height (DBH)] were chosen for salt treatments in July. These samples were grouped into five treatments with four replications per treatment. Treatments included different salt solutions at the following levels: control (CK; 0 mmol/L NaCl), 100 mmol/L NaCl, 200 mmol/L NaCl, 300 mmol/L NaCl, and 400 mmol/L NaCl. The solution volume was 3 L, and it was poured into pots all at once, followed by watering the rest of the time. A pilot study showed that the leaves were completely exfoliated after 35 days of 400 mmol/L NaCl concentration. Salt treatments lasted 28 days.

### 2.3 Measurement of leaf-specific hydraulic conductance

The original hydraulic conductance was determined using a high-pressure flow meter (HPFM-GEN3, Dynamax Inc., Houston, TX, USA) using the method of water perfusion. The HPFM system included a mechanism that pumped distilled water into specific plant sections, allowing for the determination of homologous flow rates. By analyzing the correlation between flow rate and applied pressure, the original hydraulic conductance was derived. To measure the original hydraulic conductance in shoots, a pressure of approximately 300–350 kPa was applied until a stable water flow rate was achieved. Measurements of the original hydraulic conductance in roots were determined using imposed instantly ascending pressure from 0 kPa to 500 kPa; then, the data were obtained using linear regression. All measurements were conducted for approximately 10 minutes, and the initial values were subsequently adjusted to reflect those at 25°C. This correction was necessary to account for variations in water viscosity caused by differing temperatures during each measurement.

The original hydraulic conductance of roots and shoots was determined using the aforementioned method, with specific steps outlined below. Initially, the stalk was severed from the main root at a distance of 3–4 cm above the soil surface. Subsequently, the ends of the intact roots and shoots were connected to the testing instrument to measure their original hydraulic conductance. The whole shoot system underwent *in vitro* measurement, whereas the entire root system underwent *in situ* measurement. Subsequently, the leaves were removed to derive new values, revealing the original hydraulic conductance of the stem. Finally, the original hydraulic conductance of the leaves was determined using an analog based on Ohm’s law, which utilized hydraulic pressure measurements (Alsina et al., 2011). Ultimately, the water transport efficiency of specific plant parts was quantified as leaf-specific hydraulic conductance values, which could reflect the capacity of the specific plant tissue to supply water to the evaporation surfaces, expressed as hydraulic conductance per unit leaf area (*k*, kg·s^−1^·MPa^−1^·m^−2^) (Lawren et al., 2010). These values were derived by adjusting the corrected original hydraulic conductance based on the corresponding leaf area. The hydraulic resistance ratio, derived through hydraulic segmentation, serves as a metric to reflect the hydraulic contribution of specific plant parts. This ratio is calculated by comparing the hydraulic resistance (inverse of the original hydraulic conductance) of each plant part to the overall hydraulic resistance of the entire plant, expressed as a percentage.

### 2.4 Leaf area

To determine leaf area, the number of leaves per sapling in each treatment was placed on grid paper, and the edges of the leaves were traced. The total number of square grids was counted where the leaf area occupied more than 50% of each grid. Subsequently, the area of a single grid was multiplied by the count of these grids to calculate the total leaf area.

Leaves were washed and dried completely at 80°C, and their biomass was obtained by weighing them on an electronic balance. The *SLW* values for each sapling were calculated by dividing leaf area by biomass (Becker et al., 2000). These dried leaves were preserved for ion content measurements after leaf area determination.

Fresh leaves were preserved in liquid nitrogen for subsequent physiological and biochemical parameter measurements, and their leaf area was measured using the previously described method. To calculate the residual leaf area, the dry weight and specific leaf weight (*SLW*) values were utilized, assuming a consistent SLW across all saplings. The total leaf area of the entire plant was determined by summing the three separate leaf area measurements obtained previously.

### 2.5 Gas exchange parameters

Gas determinations were taken all at once following the salt treatments. The LI-6400 portable photosynthesis system (LI-COR, Lincoln, NE, USA) was used for determining transpiration (E, mmolH_2_O·m^−2^·s^−1^), net photosynthetic rate (Pn, μmolCO_2_·m^−2^·s^−1^), and stomatal conductance (*g_s_*, molH_2_O·m^−2^·s^−1^). Water use efficiency (*WUE*, μmolCO_2_·mmolH_2_O^−1^) was determined as the ratio of net photosynthetic rate to transpiration rate. Three or four fully expanded leaves from three to four branches on the top of each shoot were used for the measurement. Gas exchange parameters in *P. euphratica* were measured at noon using a Standard Chamber, which had clear-top chamber systems and could measure the ambient photosynthetically active radiation.

### 2.6 Physiological and biochemical parameters

Physiological and biochemical parameter measurements were performed using testing kits supplied by Comin Biotechnology in China. Before weighing with an analytical balance, a 0.1-g sample was ground in liquid nitrogen. Subsequently, the sample was homogenized in a phosphate buffer with a pH of 7.8. After centrifuging the homogenate at 12,000 rpm at 4°C for 15 minutes, MDA, SOD, POD, and CAT analyses were conducted. The resulting supernatant was utilized for the determination of SOD, POD, and CAT activities, employing thiobarbituric acid chronometry, nitroblue tetrazolium, and guaiacol staining methods, respectively.

To determine MDA content, a 0.1-g sample was ground in liquid nitrogen, accurately weighed, and extracted with 3% sulfosalicylic acid in a 5-mL volume. This homogenate was centrifuged at 10,000 rpm for 10 minutes at 25°C after being placed in a centrifuge tube and extracted by vibrating in a hot water bath for 10 minutes at 95°C. Proline content was measured using acid ninhydrin colorimetry with the cooled homogenate. For this, 0.1–0.2-g samples were precisely weighed, fully ground with a small amount of distilled water, and filled to 10 mL with deionized water. After being incubated in a hot water bath for 30 minutes at 95°C, the supernatant was obtained by centrifuging at 3,000 rpm. Soluble sugar content in the supernatant was then determined using the anthrone colorimetric method.

Ion content was determined using an inductively coupled plasma–optical emission spectrometer (ICP-OES; Optima 8000, PerkinElmer, Waltham, MA, USA). Dried leaves were ground in the lab for further studies, and 0.25-g subsamples were nitrified in ceramic crucibles on an electric hot plate for 1 h at 200°C with 4 mL high-purity HNO_3_ to achieve near dryness. The crucibles with dried samples were placed in a muffle furnace and ashed for 8 h at approximately 500°C. Finally, the residuum was fully dissolved using 4 ml HCl. The solution was filled to 10 mL with deionized water for measurements of Mg^+2^, Ca^+2^, K^+^, and Na^+^ concentrations.

### 2.7 Data treatment and statistical analysis

To assess the impact of varying salinity treatments on hydraulic, gas exchange, and physiological and biochemical parameters, the analysis of variance (ANOVA) method was employed. The least significant difference (LSD) test, utilizing *post-hoc* means, was chosen for comparison. Statistical significance was determined using Pearson’s product-moment correlation at a probability threshold of *p* < 0.05. Data were represented as mean values with standard deviation and were statistically analyzed using SPSS 19.0 software. Figures were graphically represented using Origin 8.0.

## 3 Results

### 3.1 Changes in hydraulic parameters

**Figure 2:**
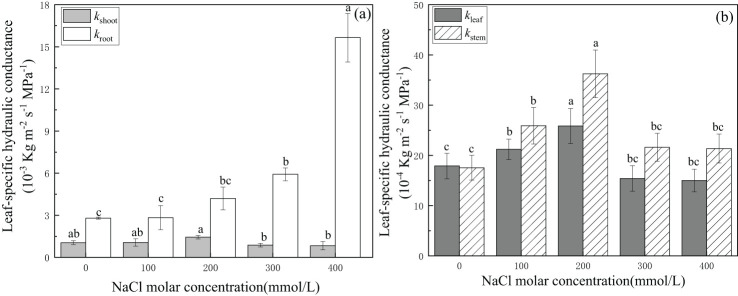
Changes in leaf-specific hydraulic conductance of the whole plant **(A)** and the aboveground parts **(B)** with different salinity concentrations. **(A)** *k_root_* (open bars) indicates leaf-specific hydraulic conductance of whole roots, and *k_shoot_* (light-shaded bars) indicates leaf-specific hydraulic conductance of whole shoots. **(B)** *k_leaf_* (light-shaded bars) indicates leaf-specific hydraulic conductance of leaves, and *k_stem_* (hatched bars) indicates leaf-specific hydraulic conductance of stems. Different lowercase letters denote significance levels based on ANOVA *post-hoc* means with least significant difference (LSD) analysis (*p* < 0.05). Data are means with standard error.

Leaf-specific hydraulic conductance of the root system increased gradually with increasing salinity concentration from CK to 300 mmol/L NaCl treatment and became significant at 1.57 × 10^−2^ kg·m^−2^·s^−1^·MPa^−1^ in the 400 mmol/L NaCl salinity treatment. Whole shoot leaf-specific hydraulic conductance increased gradually with increasing salt concentration from CK to 200 mmol/L NaCl and then decreased significantly in the 300 mmol/L NaCl treatment to 8.73 × 10^−4^ kg·m^−2^·s^−1^·MPa^−1^ or approximately 16.9% lower than the values in CK. In addition, it was approximately 20.8% lower in 400 mmol/L NaCl than that in the CK treatment (**Figure 2A**). The trend in root water transport capacity showed that roots were more adaptable than shoots of *P. euphratica* under severe salt stress.

Leaf-specific hydraulic conductance increased significantly with increasing salt concentration from CK to 200 mmol/L NaCl treatment and was 2.59 × 10^−3^ kg·m^−2^·s^−1^·MPa^−1^ in 200 mmol/L NaCl or 1.45 times the value in CK (without salinity), and then decreased significantly in 300 mmol/L NaCl. It was approximately 15.6% lower than that in the CK treatment in the salinity treatment of 400 mmol/L NaCl.

The trend in stems was the same as that in leaves. Both values in leaves and stems reached the highest value in 200 mmol/L NaCl treatment, and the values of the stem in other salt treatments were higher than those in CK. The final value of leaf-specific hydraulic conductance in the stem was 2.14 × 10^−3^ kg·m^−2^·s^−1^·MPa^−1^ or 1.22 times the value in CK in the 400 mmol/L NaCl (**Figure 2B**) treatment. This indicated that leaves and stems of *P. euphratica* could effectively enhance the efficiency of water transport within the salt concentration of 200 mmol/L NaCl.

**Figure 3:**
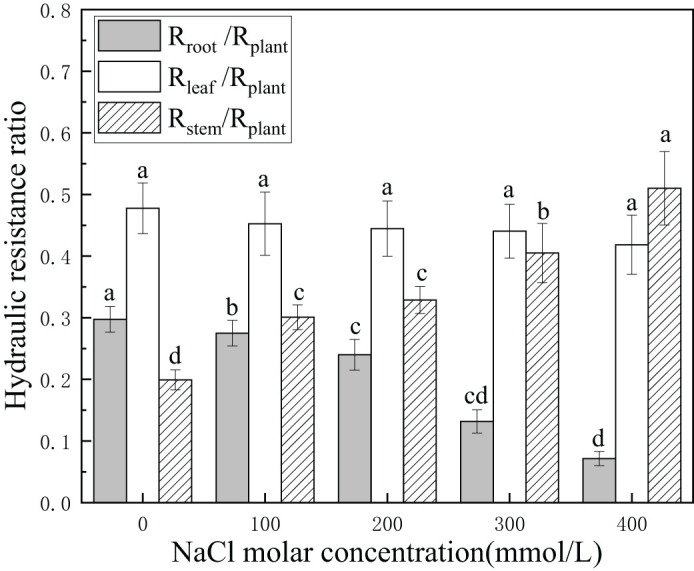
Changes in hydraulic resistance ratio with different salinity concentrations. R_root_/R_plant_ (light-shaded bars) indicates the hydraulic resistance ratio of whole roots to the whole plant, R_stem_/R_plant_ (hatched bars) indicates hydraulic resistance ratio of stems to the whole plant, and R_leaf_/R_plant_ (open bars) indicates the resistance ratio of leaves to the whole plant. Different lowercase letters denote significance levels based on ANOVA *post-hoc* means with least significant difference (LSD) analysis (*p* < 0.05). Data are means with standard error.

There was a gradual decline from 29.7% to 7.2% in the hydraulic resistance of roots relative to the plant. This suggested that the hydraulic contribution of the root system was significantly enhanced in salinity stress. The hydraulic resistance of stems initially accounted for 19.9% of that of plants. The hydraulic resistance of stems relative to the plant increased from 19.9% to 51.1% with an increase in salinity concentration. In contrast, the differences in hydraulic resistance of leaves were not statistically significant (*p* > 0.05), with values between 41.8% and 47.8%. This indicated that the hydraulic contribution of stems was significantly reduced, while that of the leaves was slowly enhanced in salinity stress. The hydraulic resistance of leaves was higher than that of stems under all salinity treatments except for 400 mmol/L NaCl (**Figure 3**). This demonstrated that leaves had the greatest hydraulic limitation in overground parts and roots with lower hydraulic limitation than the plant, playing a vital role for *P. euphratica* in salt stress. Moreover, changes in the hydraulic resistance ratio of other plant tissues ensured the stability of the hydraulic resistance ratio of leaves, which sustained a stable water supply to the evaporation surface relative to the whole plant.

### 3.2 Changes in gas exchange parameters

**Figure 4:**
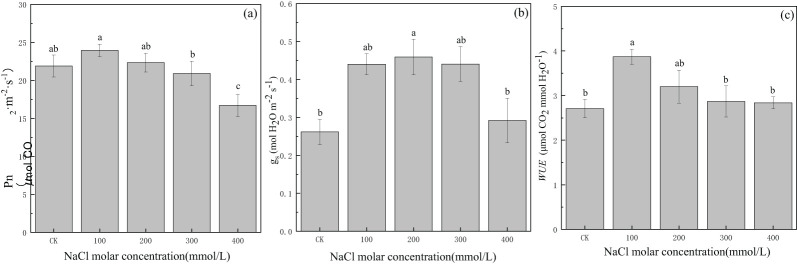
Changes in net photosynthetic rate **(A)**, stomatal conductance **(B)**, and water use efficiency **(C)** with different salinity concentrations. Lowercase letters over the bars denote significance levels based on ANOVA *post-hoc* means using least significant difference (LSD) analysis (*p* < 0.05).

The net photosynthetic rate (Pn) gradually increased from CK to 100 mmol/L NaCl treatment and was 23.95 μmolCO_2_·m^−2^·s^−1^; then, it decreased gradually with increasing salinity concentration until 16.71 μmolCO_2_·m^−2^·s^−1^ in 400 mmol/L NaCl or approximately 23.7% lower than that in CK (**Figure 4A**). Stomatal conductance (*g_s_*) in the CK treatment was 0.26 molH_2_O·m^−2^·S^−1^, and it gradually increased and then gradually decreased with increasing salinity concentration. The maximum value was 0.46 molH_2_O·m^−2^·S^−1^ in 200 mmol/L NaCl treatment, which was 1.8 times the value in CK. The values of *g_s_* in other salinity treatments were greater than those in CK (**Figure 4B**). The water use efficiency (*WUE*) value in CK was 2.71 μmolCO_2_·mmolH_2_O^−1^, and the trend in water use efficiency was the same as that in stomatal conductance. The maximum value was 3.87 μmolCO_2_·mmolH_2_O^−1^ in 100 mmol/L NaCl treatment, which was 1.4 times the value in CK. The values of *WUE* in other salinity treatments were greater than those in CK (**Figure 4C**). The increase in CO_2_ via open stomata increased carbon fixation in the blade, resulting in increased Pn; meanwhile, the increase in transpiration rate was small, and plant *WUE* increased. This suggested that the degree of salinity concentration exerted different influences on gas exchange, and photosynthesis as well as water use efficiency and stomatal conductance could be enhanced under a certain degree of salt stress for *P. euphratica*.

### 3.3 Changes in physiological and biochemical parameters

**Figure 5:**
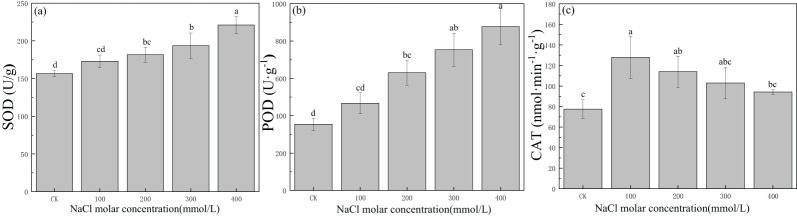
Changes in superoxide dismutase (SOD) **(A)**, peroxidase (POD) **(B)**, and catalase (CAT) **(C)** with different salinity concentrations. Lowercase letters denote significance levels based on ANOVA *post-hoc* means using least significant difference (LSD) analysis (*p* < 0.05). Data are means with standard error.

The SOD activity was 156.48 U/g in CK, and it increased to 221.07 U/g in leaves with an increase in salinity concentration, a value 1.4 times that in CK (**Figure 5A**). There also was a gradual increase from 354.37 U/g to 877.37 U/g in POD with increasing salinity concentration (**Figure 5B**). The CAT activity increased significantly at first and then gradually declined, with a maximum value of 127.81 nmol·min^−1^·g^−1^ in 100 mmol/L NaCl, which was 1.7 times the value in CK. The CAT activity in other salinity treatments was greater than that in CK (**Figure 5C**). This indicated that SOD, POD, and CAT could provide protection even under severe salt stress; meanwhile, SOD and POD could provide longer-lasting protection for *P. euphratica* with increasing salinity concentration.

**Figure 6:**
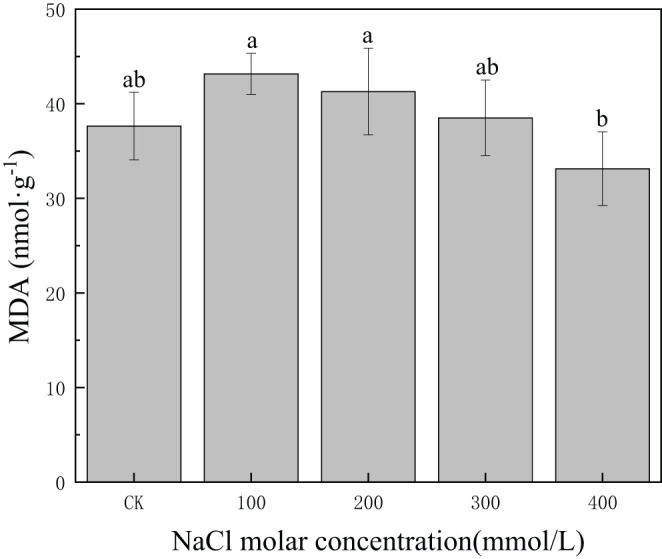
Changes in malondialdehyde (MDA) content with different salinity concentrations. Different lowercase letters denote significance levels based on ANOVA *post-hoc* means with least significant difference (LSD) analysis (*p* < 0.05). Data are means with standard error.

MDA content was 37.64 nmol/g in CK, and it increased and then decreased slowly with increasing salinity concentration. MDA content decreased to 33.13 in 400 mmol/L NaCl, but the differences in MDA content between CK and 400 mmol/L NaCl treatment were not statistically significant (*p* > 0.05) (**Figure 6**). This demonstrated that MDA content could be maintained at a low level under severe salt concentrations, reflecting relatively minor damage to cell membranes induced by salt stress.

**Figure 7:**
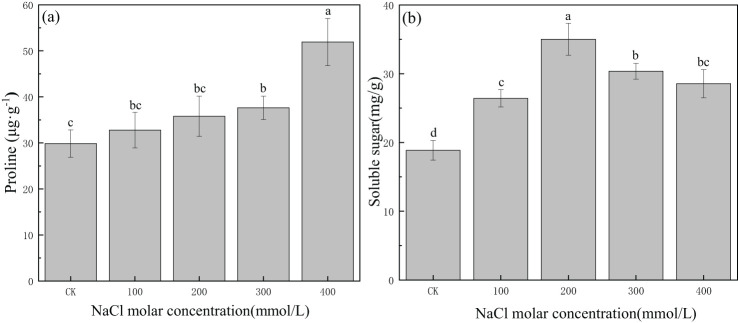
Changes in proline **(A)** and soluble sugars **(B)** with different salinity concentrations. Lowercase letters denote significance levels based on ANOVA *post-hoc* means using least significant difference (LSD) analysis (*p* < 0.05). Data are means with standard error.

Proline content was 29.85 μg/g in CK, and it continued to increase with increasing salinity concentration. Proline content increased gradually from CK to the 300 mmol/L NaCl treatment, and then it significantly increased to 51.92 μg/g in 400 mmol/L NaCl (**Figure 7A**). The soluble sugar content was 18.85 mg/g in CK, and it increased greatly and then decreased gradually with increasing salinity concentration. The maximum value was 35.01 mg/g in the 200 mmol/L NaCl treatment, which was 1.9 times the value in CK. Concentrations of soluble sugars in other salinity treatments were greater than those in CK (**Figure 7B**). This indicated that proline and soluble sugars could accumulate in *P. euphratica* and were involved in osmotic regulation under certain salinity concentrations.

**Figure 8:**
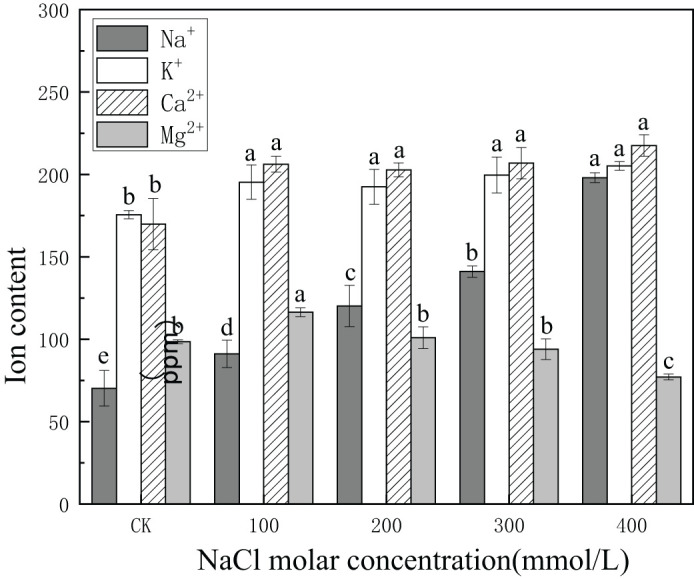
Changes in ion content with different salinity concentrations. Dark-shaded bars indicate Na^+^ concentrations, open bars indicate K^+^ concentrations, hatched bars indicate Ca^+2^ concentrations, and light-shaded bars indicate Mg^+2^ concentrations. Different lowercase letters denote significance levels based on ANOVA *post-hoc* means with least significant difference (LSD) analysis (*p* < 0.05). Data are means with standard error.

The concentration of Na^+^ increased from 70.3 ppm to 198.1 ppm or 2.8 times the values in CK, with increasing salinity concentration. The concentration of K^+^ increased with increasing salinity concentration from 175.58 ppm in CK to 205.15 ppm in 400 mmol/L NaCl or 1.17 times the value in CK. The concentration of Ca^+2^ increased from 169.88 ppm in CK to 217.58 ppm in 400 mmol/L NaCl or 1.3 times the value in CK. Concentrations of Ca^+2^ and K^+^ increased in all salinity treatments. By contrast, Mg^+2^ content in CK was 98.51 ppm, increasing significantly to 116.38 ppm in 100 mmol/L NaCl, and then gradually declining to 77.11 ppm in 200 mmol/L NaCl, which was lower than that in CK (**Figure 8**). This demonstrated that Na^+^, K^+^, and Ca^+2^ could continuously accumulate and were involved in osmotic regulation in *P. euphratica* with increasing salinity concentrations.

## 4 Discussion

### 4.1 Adaptability of plant water transport to salt stress

The leaf-specific hydraulic conductance of roots in *P. euphratica* improved under salinity stress. Meanwhile, the leaf-specific hydraulic conductance of specific tissues (i.e., whole shoots, leaves, and stems) improved under mild and moderate salt stress (less than 200 mmol/L NaCl), and these parts could maintain hydraulic conductance per leaf area under severe and without salt stress. The findings suggested that *P. euphratica* exhibited a distinct self-regulation ability under mild to moderate salt-stress conditions; however, its self-adaptive and regulatory capacity for water transport diminished under severe salt stress (more than 300 mmol/L NaCl). This also showed that *P. euphratica* could maintain water transport efficiency, which ensured its survival in a salt-stress environment.

Roots are the main tissues in plants that absorb both water and nutrients from the soil. Roots experience water stress before other plant systems do, and the hydraulic characteristics of roots exhibited higher values than shoots of *P. euphratica* in salinity stress, demonstrating that the roots were more sensitive to salt stress than aboveground parts of *P. euphratica*. Therefore, roots of *P. euphratica* could significantly enhance water transport efficiency under salt stress. These results were similar to those in mangrove species, in which very high salt concentrations could help regulate hydraulic conductance in the pit membranes of vessels (Lopez-Portillo et al., 2005). However, the water transport efficiency of roots in some tree species was reduced under salt-stress conditions (Rodríguez-Gamir et al., 2012; Calvo-Polanco et al., 2014). This was presumably owing to the specific hydraulic characteristics of tree species in arid regions. Previous studies suggested that the hydraulic resistance of roots in different tree species contributed between 20% and 90% to the whole plant under diverse conditions (Parent et al., 2009). This showed that the regulation of the root hydraulic contribution depended on both the living conditions and tree species. Hydraulic resistance of the root provided less than one-third of the internal hydraulic limitation in *P. euphratica* and gradually decreased with increasing salt stress. These results provided evidence that the root system was critical for water transport in salinity adaption for *P. euphratica*.

In addition, hydraulic limitation in stems progressively increased with salt stress, indicating that salinity stress may have a larger impact on changes in water transport efficiency in stems than in leaves (Li et al., 2019). It was likely that stems still had a water-transfer function under salt stress, while stems lost water transport and regulation function under extreme drought conditions due to the death of the aboveground parts. Furthermore, leaves contributed almost half of the plant’s internal resistance, and that remained relatively stable in salt stress. This illustrated that *P. euphratica* leaves maintained a stable water supply relative to the whole plant. Under mild and moderate salt stress, increased water transport efficiency and enhanced water transfer process in all parts helped *P. euphratica* to improve its salinity-resistant capability. These results indicated that the water transfer process is elastic in relatively advantageous environments while a bold strategy in relatively unfavorable environments.

### 4.2 Adaptability of water utilization in plants to salt stress

Photosynthesis is a basic physiological process, the source of organic matter synthesis and biomass accumulation, which is also one of the main metabolic processes affected by salinity in plants (Zhang et al., 2014; Diao et al., 2014; Xu et al., 2015). Photosynthesis in *P. euphratica* was enhanced under relatively mild and moderate salinity stress but weakened under severe salinity stress. This showed that exposure to high salt concentrations diminished the water absorption capacity and hindered growth in certain plant species by compromising photosynthetic efficiency and disrupting metabolic processes (Deinlein et al., 2014; Duan et al., 2018; Batista et al., 2019). These results were similar to those in *Tetrastigma hemsleyanum*, *Alhagi pseudalhagi*, and *Desmostachya bipinnata* (Asrar et al., 2017; Wungrampha et al., 2018; Guo et al., 2018). The acceleration of photosynthesis could offer more energy for transpiration and stomatal conduction with mild interference under lower salinity concentration; in contrast, the reduction in the rate of photosynthesis was due to diminished adaptability to the environment with severe interference under higher salinity concentration in *P. euphratica* (Yang et al., 2021).

Leaves are the main parts used in plant transpiration, and previous studies have shown a decrease in stomatal conductance and water use efficiency with stomatal closure, which further inhibits carbon fixation by reducing CO_2_ availability in the leaves with an increase in salinity concentration (Duan et al., 2018; Liu et al., 2014; Lawson and Matthews, 2020). Both water use efficiency and stomatal conductance of *P. euphratica* were enhanced under lower salinity concentrations and weakened in severe salt stress. In this study, we found that under mild and moderate salt stress, the stomatal conductance of *P. euphratica* did not change significantly, which is attributed to the efficient water transport in stems and the sufficiency of water in leaves.

Stomatal conductance, the main regulator of water flow in plants in the short term, could be affected by increased water transport efficiency by magnifying the hydraulic signal (Liu et al., 2014; Pan et al., 2016). With the increase in salt concentration, the stomatal conductance of *P. euphratica* leaves decreased, and stomatal closure limited water loss for maximum CO_2_ assimilation through a short-term response; this led to a decrease in leaf transpiration rate and an increase in water utilization, thus improving the adaptability of plants to salt stress.

However, this adaptability is limited. With the extension of salt stress, plant water imbalance was aggravated, photosynthetic capacity continued to decline, and it finally inhibited the water use efficiency of *P. euphratica*. As a result, *P. euphratica* could enhance its water use efficiency coupled with increasing water transport efficiency when encountering a certain extent of salinity threat. The strengthened water use process coupled with the enhanced water transfer process of *P. euphratica* experiencing mild to moderate salt stress formed an increase in hydraulic capacity.

### 4.3 Adaptability of water storage in plants to salt stress

Salt stress induces excessive excitation energy by exposing chloroplasts, which could promote ROS generation and oxidative stress (Ray et al., 2012). Enhanced peroxidase enzymes enable plants to defend against oxidative stress and dissipate harmful molecules, indicating increased production of ROS (Demidchik, 2015; Rajput et al., 2015). POD and CAT are examples of enzymes that take part in catalyzing the conversion of H_2_O_2_ to H_2_O and O_2_, followed by the enzyme SOD converting ROS to H_2_O_2_, which promoted the scavenging of ROS (Liu et al., 2015). The activities of SOD and POD in *P. euphratica* were enhanced with salinity stress, while CAT activity was enhanced under low salt stress, which indicated that SOD and POD provided relatively long-lasting protection than CAT under salt stress.

The generation of ROS is highly harmful to plant cells, particularly to membrane integrity due to the acceleration of disruption membrane by the arachidonic acid decomposition and MDA generation with the extension of salt stress (Ray et al., 2012; AbdElgawad et al., 2016). Moreover, the less de-structured the membranes, the lower the MDA generation, which is an index that represents membrane functional integrity (Bezerra-Neto et al., 2022). Under salt-stress conditions, the MDA content in *P. euphratica* remained stable, preventing membrane damage. Prior studies on salt-tolerant species have demonstrated that low MDA levels could significantly contribute to plants’ adaptability and tolerance to saline environments (Sergio et al., 2012). Higher activities of SOD, POD, and CAT, and low MDA content observed in salinity-treated plants indicated that *P. euphratica* had a higher ability to eliminate ROS and establish a mechanism of protection against oxidative damage as concluded in previous studies (Rajput et al., 2015). This showed that regulation of the antioxidant enzyme clearance system, including SOD, POD, CAT, and MDA, could help plant cells achieve effective water storage function under salinity stress.

Plants formed adaptations to soil salinity that alleviate the disadvantageous effects of ionic and osmotic stresses by producing osmotic compounds and regulating ion transfer, sustaining tissue metabolic activity and water storage in response to slowly imposed salt-induced dehydration (Zelm et al., 2020). Production of cytoplasmic organic compounds such as amino acids and sugars eventually leads to the restoration of cellular homeostasis, detoxification, and therefore survival under stress (Parihar et al., 2015). Soluble sugars altered by salinity serve as signaling molecules and interact with hormones as part of the signaling network in plants under stress, which also may maintain cell turgor pressure and stabilize proteins, membranes, and enzyme activity (Per et al., 2017; Meena et al., 2019).

Soluble sugars and proline content in *P. euphratica* increased with salinity stress, which may be partly explained by the strengthening of enzyme activity under salt stress. Proline is an important component of aquaporins, the activity of which could affect the water transfer process by altering the physiology of branches during osmotic stress, and also plays a vital role in osmotic adjustment in plants under saline stress (Maurel et al., 2010; Romero et al., 2012). Proline content in *P. euphratica* increased with salinity stress, which may be partly explained by the strengthening of water transport efficiency of shoots under mild and moderate salt stress. This indicates that the accumulation of soluble sugars and proline in *P. euphratica* can increase osmoregulation capability, mitigate salt-induced damage, and ensure the growth and survival of *P. euphratica* subjected to salinity conditions.

In summary, our results suggested that the production of total soluble sugars and proline in leaves may be related to saline stress tolerance and osmotic adjustment, which concurred with the findings of prior studies (Moukhtari et al., 2020). Except for osmotic stress, ionic stress is triggered by superfluous salts, which leads to cell membrane stability and physiological activity in plants (Tavakkoli et al., 2011; Liang et al., 2018; Isayenkov and Maathuis, 2019). Homeostasis of the plant is crucial to guarantee osmotic adjustment, which relies on inorganic osmotic regulators to protect from toxicity in high salinity (Bezerra-Neto et al., 2022). In addition to Na^+^, the content of other main inorganic osmolytes K^+^ and Ca^+2^ increased in *P. euphratica* with salinity stress. Meanwhile, Mg^+2^ content increased in response to low salt stress but decreased under conditions of high salt stress.

Our research suggested that the K^+^ accumulation in the plant would probably be a strategy for salinity tolerance. Past research has also shown that plants with greater tolerance to salinity often exhibit slightly higher K levels (Pompelli et al., 2010; Silva et al., 2015; Borrelli et al., 2017). However, there could be such a risk that the exchange of Ca combined with the plasma membrane because of the high external salt concentrations is the principal factor in salinity stress (Ottow et al., 2005). The results showed that Ca accumulation in leaves increased slightly with salinity stress, and that was probably related to the accumulation of proline, which could improve ionic homeostasis (de Freitas et al., 2019; Rady et al., 2016). In addition, K and Mg are cofactors for various enzymes in photosynthesis (Tränkner et al., 2018; Bezerra-Neto et al., 2022), which may partly explain the enhanced photosynthesis under low salt conditions. In a salt environment, a momentum of expanding pressure in plant cells exists via osmoregulation of organic compounds and accumulation of inorganic osmolytes, in addition to an antioxidant enzyme scavenging system for maintaining a certain moisture content. As a result, water storage in leaves decreases reliance on soil moisture content by alleviating water stress in leaves (Simonin et al., 2009; Ishii et al., 2014). Thus, the water retention process coordinated with the water transfer process and water use process, which played a vital part in the stress resistance of plants.

## 5 Conclusions

We showed coordination of water processes in *P. euphratica* under salt stress by analyzing gas exchange, hydraulic, and physiological and biochemical parameters in this riparian tree species. *P. euphratica* could improve its water transport efficiency in salinity stress, and roots contributed more to the water transfer process than the aboveground parts. In addition, *P. euphratica* could improve water use efficiency and increase water transport efficiency under mild and moderate salt stress but not under severe salt stress. Furthermore, *P. euphratica* could increase osmoregulation by accumulating organic compounds and inorganic osmolytes and strengthen the protection function of the antioxidant enzyme scavenging system in the water storage process to coordinate with increased water transport efficiency and water use efficiency. As a result, water transport, utilization, and storage processes worked together to promote plant survival in salinity.

Plants strive to increase water transport efficiency and water use efficiency and maintain water storage ability in mild and moderate salt stress. Plants can reduce stress-induced damage and achieve growth using defense and protection mechanisms in low unfavorable salt-stress environments. The effect of salt toxicity on physiological and metabolism processes involving water processes in plants depends on the salt content. The changes reflect adaption in the strategy involving water processes in riparian plants residing in diverse salt-stress environments.

Riparian plants can acclimatize to adverse conditions through performance improvement and adaptive functional characteristics. The alterations in water processes within riparian plants under varying salt-stress environments provide a scientific foundation for the restoration and preservation of riparian forests. Additional anatomical studies on *P. euphratica* grown in the wild combined with the local specific hydrological environment will enhance our comprehension of its internal adaptation mechanisms under saline conditions.

## Data Availability

The original contributions presented in the study are included in the article/supplementary material. Further inquiries can be directed to the corresponding author.
